# New Mouse Model Suggests That Some Neuroendocrine Tumors May Originate From Neural Crest–Derived Cells

**DOI:** 10.1016/j.jcmgh.2022.08.005

**Published:** 2022-09-06

**Authors:** D. Mark Pritchard

**Affiliations:** Department of Molecular and Clinical Cancer Medicine, Institute of Systems, Molecular and Integrative Biology, University of Liverpool, The Henry Wellcome Laboratory, Liverpool, United Kingdom

Gastroenteropancreatic neuroendocrine neoplasms (GEP-NENs) are a heterogeneous group of tumors whose incidence has increased considerably over the past 50 years, probably largely because of greater awareness.^1^ Several phase 3 clinical trials also have led to significant recent improvements in treatment, especially for those patients who have metastatic well-differentiated low grade neuroendocrine tumors (NETs).^2^ However, additional developments in this field currently are being hampered by a paucity of in vitro and in vivo models that accurately recapitulate disease pathogenesis and therefore provide platforms for testing potential new treatments.

One key gene that is mutated in GEP-NENs is the *MEN1* gene. Inherited inactivation of the *MEN1* locus leads to multiple endocrine neoplasia (MEN1) syndrome, in which people develop tumors in the pancreas, pituitary gland, and parathyroid gland.^3^ However, *MEN1* mutations also are common in sporadic GEP-NENs, especially those that arise in the pancreas.^4^ Heterozygous deletion of *Men1* in mice was shown almost 20 years ago to recapitulate some of the phenotypic features of human MEN1.^5^ More recently, Sundaresan et al^6^ also showed that conditional deletion of *Men1* from the gastrointestinal tract using the *Villin Cre* transgene on a somatostatin-null genetic background resulted in the development of gastric NETs. However, these prior investigations did not investigate the potential cell of origin of NETs.

In the current issue of *Cellular and Molecular Gastroenterology and Hepatology*, Duan et al^7^ have built on preliminary findings from their lab that were described in the paper by Sundaresan et al^6^ to investigate whether some GEP-NENs develop from reprogrammed neural crest–derived cells rather than from endoderm-derived enteroendocrine cells. They conditionally deleted *Men1* from glial fibrillary acid protein (GFAP)-expressing glial cells in mice and showed a phenotype closely resembling human MEN1. The mice developed NENs in the pancreas and pituitary gland. Most of the pancreatic tumors were well-differentiated NETs and the pituitary tumors were predominantly prolactinomas. Intriguingly, the pituitary tumors developed much more commonly in female mice, consistent with the sex difference observed in human MEN1. The reasons for this sex difference, however, have not yet been confirmed. In addition, the mice developed gastric neuroendocrine hyperplasia. However, this was not secondary to gastrinoma development because it was associated with parietal cell atrophy rather than with parietal cell hyperplasia (which is observed in MEN1 patients who have gastrinomas). Glial-directed *Men1* deletion, however, was insufficient to cause NET development in the stomach. Importantly, the development of neuroendocrine neoplasia/hyperplasia in *GFAP*^*ΔMen*^^*1*^ mice was associated with loss of the GFAP-restricted lineage, suggesting that removal of menin resulted in glial cell reprogramming. A similar phenomenon also was found after small interfering RNA–induced knockdown of *Men1* expression in a GFAP-expressing rat enteric glial cell line. The investigators next conducted RNA sequencing transcriptomic analysis using tissues from *GFAP*^*ΔMen1*^ transgenic mice and, among a number of observations, found that Hedgehog signaling appeared to regulate NET development. They therefore deleted *Kif3a*, a ciliary motor protein required for Sonic Hedgehog signaling in *GFAP*^*ΔMen*^^*1*^ mice and showed an attenuation of gastric neuroendocrine hyperplasia.

Limitations of the *GFAP*^*ΔMen*^^*1*^ mouse include that it is a constitutive model and that GFAP expression is not restricted to enteric glial cells, especially during animal development. To address these limitations, the investigators also deleted *Men1* from *Sox10*–expressing cells (which are present in the neural crest and maintained in glial lineages) and showed a similar phenotype with pancreatic NET development. However, *Sox10*^*ΔMen1*^ mice did not develop pituitary tumors and gastric neuroendocrine hyperplasia/neoplasia was observed only occasionally. This second mouse model therefore supports the hypothesis that neural crest–derived cells may be the cell of origin of some NETs. However, confirmatory lineage tracing coincident with inducible deletion of *Men1* using *CreERT* technology will be needed before this can be definitely confirmed.

In conclusion, Duan et al^7^ have shown that deletion of *Men1* from GFAP-expressing glial cells led to loss of this cell lineage, its reprogramming to an endocrine phenotype, and NET development in the pancreas and pituitary. These findings raise the possibility that some NENs arise from the reprogramming of neural crest–derived cells rather than from enteroendocrine cells. Considering the diversity of anatomic sites and biological/clinical behaviors of GEP-NENs, it is unlikely that these tumors all have the same cell of origin. However, if it is possible to determine which subcategories of NET develop via this pathway, this may have implications for treatment and/or prognosis. One future direction will be to evaluate the consequences of inducible *Men1* deletion in *GFAP*- or *Sox10*–expressing cells. Similar approaches also could be used to investigate the consequences of deleting other key genes involved in NET development, such as *DAXX* or *ATRX*, as well as the effects of deleting *Men1* from other specific cell types. The development of a suite of such in vivo models will improve our understanding of GEP-NEN pathogenesis and hopefully eventually lead to improvements in treatment and patient survival.
